# The Interaction Among Effector, Regulatory, and Tγδ Cells Determines the Development of Allergy or Tolerance to Chromium

**DOI:** 10.3390/jcm14041370

**Published:** 2025-02-19

**Authors:** Magdalena Zemelka-Wiacek

**Affiliations:** Department of Clinical Immunology, Faculty of Medicine, Wroclaw Medical University, 50-367 Wrocław, Poland; magdalena.zemelka-wiacek@umw.edu.pl; Tel.: +48-506-224-077

**Keywords:** allergic contact dermatitis, chromium, chromium allergy, hapten, hypersensitivity, T regulatory cell, Tγδ

## Abstract

**Background/Objectives**: Chromium, a common environmental and occupational sensitizer, frequently induces allergic contact dermatitis (ACD). This study investigates the role of CD4^+^ (T helper), CD8^+^ (T cytotoxic), regulatory (Tregs: CD4^+^CD25^+^ and CD8^+^CD25^+^), and gamma delta (Tγδ) T cells in chromium tolerance versus hypersensitivity. **Methods**: Six chromium-allergic patients and six healthy controls were recruited, confirmed via patch testing. Peripheral blood mononuclear cells (PBMCs) were isolated and cultured, with chromium exposure and proliferation assays conducted. Specific T cell subtypes were isolated and analyzed for chromium-specific proliferative responses, cytokine production, and metabolic activity. **Results**: Chromium-allergic individuals exhibited broad proliferation across PBMC and T cell subsets, contrasting with restricted responses in controls. Treg cells in healthy subjects effectively suppressed T cell proliferation in response to chromium, while allergic individuals showed unmodulated T cell activity, indicative of impaired regulatory function. Cytokine analysis revealed elevated IL-2 and TNF-α but absent IL-10 in allergic patients. Metabolic assessments showed higher glycolytic activity in Tregs of healthy controls, suggesting enhanced regulatory potential. **Conclusions**: These findings highlight the importance of balanced effector and regulatory T cell interactions for chromium tolerance. Dysregulated Treg and Tγδ cell functions in allergic individuals may contribute to hypersensitivity, with implications for targeted therapeutic strategies to restore immune balance and reduce allergic responses in chromium-sensitive patients.

## 1. Introduction

Allergic contact dermatitis (ACD) to metals, particularly chromium, presents a significant immunopathological challenge, impacting individuals through both environmental and occupational exposures [1,2]. Although most people come into contact with ubiquitous low-molecular weight chemicals, only some develop ACD. This suggests that, under physiological conditions, the skin has regulatory mechanisms that protect against immunopathological reactions to haptens. Such mechanisms were documented in animal models [3,4]. Chromium allergy affects approximately 1% of the general population in Europe, with higher prevalence rates observed among certain occupational groups due to increased exposure. Chromium allergy severely affects patients’ quality of life, causing chronic itching, pain, and visible skin lesions, which can lead to social and occupational limitations, especially for those frequently exposed to the metal [5,6].

Chromium (Cr), found ubiquitously in materials such as cement, bleaches, detergents, leather products, cosmetics, paints, or tattoo inks, is a potent sensitizer known to elicit chronic, persistent immune responses that are more intense compared to other metal allergies [7,8,9,10,11]. Chromium ions (mainly hexavalent chromium (Cr VI)) penetrate the epidermal barrier, subsequently undergoing reduction to trivalent chromium (Cr III) and binding covalently to skin proteins, thereby forming hapten–protein complexes that are immunologically active [12,13,14]. These neoantigenic complexes are processed by antigen-presenting cells (APCs), leading to T cell activation, NLRP3 inflammasome, and a robust pro-inflammatory cascade, characteristic of hypersensitivity type IVa [15,16,17].

Despite the prevalence of metal allergies, diagnostic approaches are limited. The current standard, the chromium patch test (PT), lacks precision and sensitivity, leading to diagnostic ambiguity and requiring experienced clinical interpretation [18,19]. Metal allergies, including chromium, can also induce immune-mediated hypersensitivity reactions to implants, potentially leading to chronic inflammation, implant failure, and compromised outcomes in joint replacement surgeries [20,21,22]. Consequently, there is an urgent demand for refined diagnostic biomarkers that can enable the stratification of hypersensitivity phenotypes and, by extension, facilitate targeted interventions and individualized treatment regimens.

CD4^+^ (T helper), CD8^+^ (T cytotoxic), and T gamma delta (Tγδ) cells play distinct yet interconnected roles in type IVa hypersensitivity, a cell-mediated immune response occurring 24–72 h post antigen exposure. CD4^+^ T helper cells, particularly the Th1 subset, are pivotal, producing interferon-gamma (IFN-γ) and tumor necrosis factor-alpha (TNF-α) to activate macrophages and amplify inflammation. CD8^+^ cytotoxic T cells contribute by directly lysing antigen-presenting cells and releasing pro-inflammatory cytokines, exacerbating tissue damage. γδ T cells, bridging innate and adaptive immunity, respond rapidly to non-peptide antigens without MHC restriction. They produce interleukin-17 (IL-17) and IFN-γ, recruiting neutrophils and promoting inflammation, but also IL-10, tumor necrosis factor beta (TGF-β) [23,24,25]. CD4^+^CD25^+^ and CD8^+^CD25^+^ regulatory T cells (Tregs) are crucial modulators in ACD. These cells maintain immune homeostasis by suppressing effector T cell responses through cytokine production, such as IL-10 and TGF-β, and cell contact-dependent mechanisms. CD4^+^CD25^+^ Tregs are known to inhibit the proliferation of CD4^+^ and CD8^+^ effector T cells. Similarly, CD8^+^CD25^+^ Tregs contribute to immune tolerance by controlling cytotoxic responses. In healthy individuals, these Tregs effectively suppress the immune response to haptens/allergens, preventing overt inflammation [26,27,28]. However, in ACD patients, a dysfunction in these regulatory cells—characterized by impaired cytokine secretion or insufficient suppression of effector T cells—leads to excessive immune activation and the characteristic symptoms of hypersensitivity. This complex interaction underscores their critical roles in conditions such as ACD and hypersensitivity to metals.

The aim of this article is to investigate the presence and role of CD4^+^ and CD8^+^ and effector cells, CD4^+^CD25^+^ and CD8^+^CD25^+^ regulatory cells, and lymphocytes Tγδ, and to assess how the balance of these cell types contributes to chromium tolerance in healthy individuals compared with patients who exhibit chromium allergy.

## 2. Materials and Methods

### 2.1. Participants

Six patients with confirmed chromium allergy (aged 25–42 years; four women and two men) were recruited for the study. The selection aimed to minimize age-related confounding factors and ensure comparability between allergic and non-allergic participants. Chromium allergy was verified through the patch test using the standardized European Baseline Series (Chemotechnique Diagnostics, Vellinge, Sweden), which includes potassium dichromate VI (K_2_Cr_2_O_7_) at a concentration of 0.5%. The test was conducted by applying allergen-infused patches (IQ Ultra, Chemotechnique Diagnostics) to the participants’ backs, where they remained for 48 h. Readouts were performed at both 48 and 72 h post-application to observe any delayed hypersensitivity reactions, with positive responses confirming chromium sensitivity in these patients. Additionally, six healthy subjects (aged 23–40 years; four women and one man) with no history of contact allergies underwent the same patch test and displayed negative responses, confirming their suitability as controls.

### 2.2. Blood Samples

A total of 50 mL of blood was collected from each participant to isolate peripheral blood mononuclear cells (PBMCs), using K_2_EDTA tubes (BD Vacutainer^®^ 367525, Franklin Lakes, NJ, USA). Blood samples were processed immediately after collection to ensure the fresh isolation of PBMCs, preserving cell viability and functionality for subsequent immunological analysis.

### 2.3. Cell Purification

Peripheral blood mononuclear cells (PBMCs) were isolated from fresh blood samples using density gradient centrifugation with Ficoll-Paque Premium (Cytiva, Marlborough, MA, USA, 17-5442-03), according to the manufacturer’s protocol. Following centrifugation, PBMCs were carefully collected from the interface layer, washed extensively with phosphate-buffered saline (PBS), and resuspended in RPMI 1640 medium (Merck, Rahway, NJ, USA) prepared with 2 mM L-glutamine, 1 mM sodium pyruvate, 1% nonessential amino acids, 100 U/mL penicillin, and 100 µg/mL streptomycin (all obtained from Merck). Additionally, 5% fetal bovine serum (FBS) was added to support cell viability and stability, ensuring optimal conditions.

PBMCs were incubated in culture wells (6 × 10^6^ cells/mL, 6 mL per well) at 37 °C for 2 h to allow adherence, suspended in supplemented RPMI. After incubation, the non-adherent cells were harvested for T cell purification, while the adherent cells were treated with 0.2% EDTA for six minutes, detached using a cell scraper, and subsequently utilized as antigen-presenting cells (APCs) for functional assays. The APCs and T cells from the same donor were used to avoid allogeneic immune responses.

For cell subset separation, the T cell fraction was processed using immunomagnetic separation with MACS MicroBeads (Miltenyi Biotec, Bergisch Gladbach, Germany) following the manufacturer’s instructions. Initially, CD4^+^ and CD8^+^ T cells were isolated from the PBMC population using CD4 MicroBeads, human (Miltenyi Biotec, 130-045-101), and CD8 MicroBeads, human (Miltenyi Biotec, 130-045-201), respectively. Following initial isolation, CD4^+^ and CD8^+^ T cells were further fractionated based on CD25 expression. This secondary separation used CD25 MicroBeads, human (Miltenyi Biotec, 130-092-983), to distinguish CD4^+^CD25^+^ and CD4^+^CD25^−^ as well as CD8^+^CD25^+^ and CD8^+^CD25^−^ subsets. Tγδ cells were selectively isolated from the PBMCs using the TCR γ/δ^+^ T Cell Isolation Kit, human (Miltenyi Biotec, 130-092-892). All separations were performed using the MiniMACS™ Separator and MS Columns (Miltenyi Biotec, following the manufacturer’s protocol to ensure high purity and cell viability.

### 2.4. Proliferation Assay

A proliferation assay was conducted using the CellTrace CFSE Cell Proliferation Kit (Thermo Fisher, C34554, Waltham, MA, USA) to assess T cell division according to the manufacturer’s protocol. CFSE (Carboxyfluorescein diacetate succinimidyl ester) is a fluorescent dye used to track cell proliferation. It is excited at a wavelength of approximately 492 nm and emits fluorescence at around 517 nm, which corresponds to green light. The fluorescence signal is typically detected using the fluorescein sothiocyanate (FITC) channel. As the cells divide, the dye is equally distributed between daughter cells, leading to a progressive decrease in fluorescence intensity with each cell division. Thus, highly proliferative cells exhibit lower CFSE fluorescence, while less proliferative cells retain higher fluorescence intensity.

PBMCs were stained with CFSE (carboxyfluorescein diacetate succinimidyl ester) dye, which binds covalently to intracellular amines, providing a fluorescent signal that dilutes with each cell division, enabling the tracking of cell proliferation over time. In the presented graphs, the mean fluorescence intensity of CFSE was used to quantify the cells’ division. The gating strategy for CFSE-positive cells was established using unstained control cells, ensuring a clear distinction between CFSE-positive and CFSE-negative populations (cells underwent exactly the same procedure, including incubation; however, they were not stained with CFSE) (Supplementary Figure S1)

Following CFSE labeling, PBMCs (250,000 cells/well) or purified T cell subsets (200,000 cells/well) were plated in 96-well U-bottom plates with autologous adherent cells (150,000 cells/well) as APCs. Cells were cultured in a supplemented RPMI medium with 5% FBS. For some experiments, different numbers of cells were used (described below).

K_2_Cr_2_O_7_ at a concentration 30 µg/mL (Merck) was added to selected wells to stimulate proliferation, while the control wells contained the medium only. After incubation for 5 days at 37 °C with 5% CO_2_, cell proliferation was analyzed by flow cytometry, with CFSE fluorescence intensity used to quantify cell division rates across different T cell subsets. The concentration of K_2_Cr_2_O_7_ used in this study was selected based on experimental considerations rather than environmental exposure levels. While no standardized data are available for chromium, the chosen concentration was inspired by multiple in vitro studies on nickel and was further established through preliminary experiments to ensure an optimal balance between eliciting an immune response and maintaining cell viability.

### 2.5. Cytokine Analysis

Cytokine levels in cell culture supernatants were quantified using the BD Cytometric Bead Array (CBA) Human Th1/Th2/Th17 Cytokine Kit (BD Biosciences, # 560484, Franklin Lakes, NJ, USA). This kit enables the simultaneous measurement of multiple cytokines, including IL-2, IL-4, IL-6, IL-10, TNF, IFN-γ, and IL-17A, within a single sample. The assay was conducted according to the manufacturer’s protocol (Supplementary Figure S2). Briefly, capture beads conjugated with specific antibodies for each cytokine incubated with the samples, allowing cytokines to bind to their respective beads. Subsequently, a PE-conjugated detection reagent was added to form sandwich complexes. The samples were then analyzed using flow cytometry, and data were interpreted with the BD FCAP Array™ software v3.0 to determine cytokine concentrations.

### 2.6. Mitochondrial Analysis

Mitochondrial function was assessed using the Seahorse XF HS Mini Analyzer (Agilent Technologies, Santa Clara, CA, USA) in combination with the XFp Cell MitoStress Test Kit and XFe suspension culture microplate. The assay was conducted following the manufacturer’s protocol. Prior to the test, the medium was replaced with DMEM (pH 7.4) supplemented with pyruvate, glucose, and glutamine, as recommended by Agilent Technologies. Oxygen consumption rate (OCR) was measured in real-time under basal conditions and in response to mitochondrial inhibitors: 1.5 μM oligomycin (Oligo) to inhibit ATP-linked respiration, 1.5 μM FCCP (carbonyl cyanide-p-trifluoromethoxyphenyl-hydrazone) to assess maximal respiration, and a mixture of 0.5 μM rotenone and antimycin A (Rot/AA) to inhibit complex I and III and measure non-mitochondrial respiration.

The experimental setup included an 8-well cartridge with two background control wells and two sets of triplicates for both control and treatment conditions, enabling consistent comparison between the treated and untreated groups.

### 2.7. Statistical Analysis

As the data were too scarce to check their consistency with the normal distribution, only the non-parametric tests were used. Statistical analysis was performed using the non-parametric Mann–Whitney U test with GraphPad Prism software (version 10.2.3), considering results statistically significant at a *p*-value of less than 0.05.

## 3. Results

### 3.1. Patch Test (PT)

All six patients in the chromium allergy group had a positive PT for potassium dichromate. Among them, two patients showed a reaction of (+), three showed (++), and one exhibited (+++). Among them, some presented other allergies in addition to Cr allergy, five patients were sensitized to nickel sulfate, two to cobalt, neomycin, and fragrance mix. One patient also showed sensitivity to benzocaine, nail enamel, formaldehyde, and thiuram mix. In contrast, all six control subjects without ACD had a negative PT for potassium dichromate, confirming the absence of chromium sensitivity in the control group.

### 3.2. Proliferation of PBMC Subpopulations in Response to Chromium Exposure

**Figure 1:**
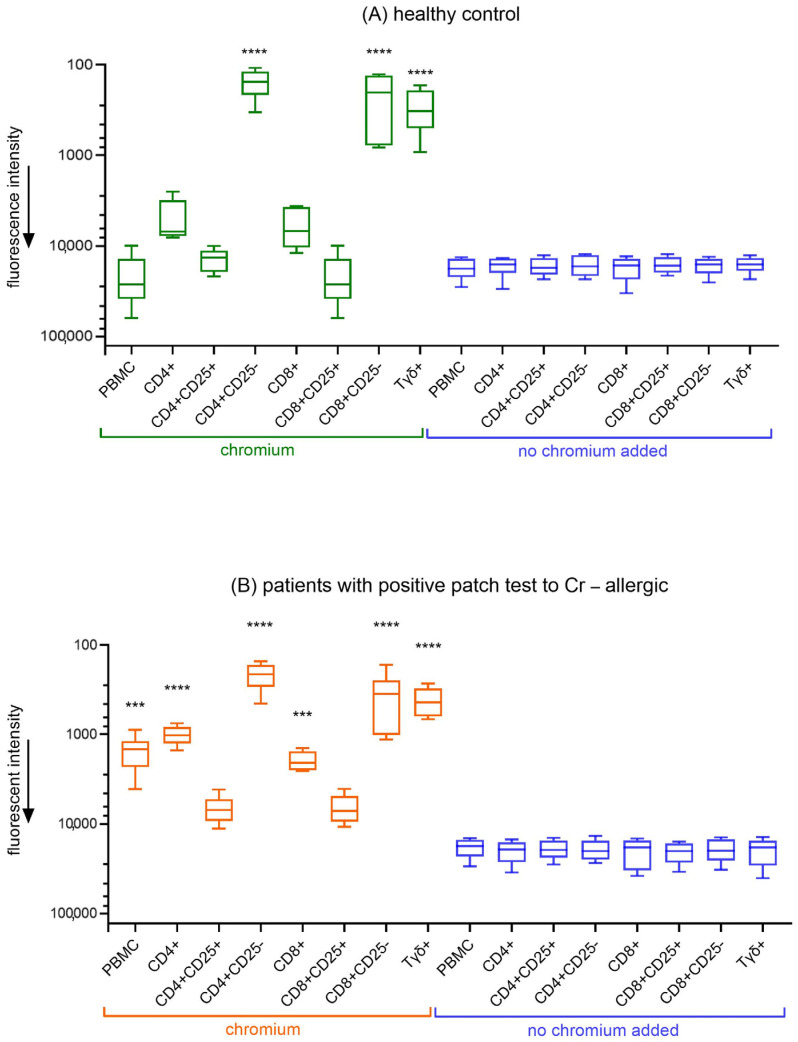
In vitro proliferation of PBMC subpopulations in response to chromium exposure. Peripheral blood from (**A**) healthy controls (HC) and (**B**) chromium-allergic individuals (Cr) was separated into T cell subpopulations, treated in vitro, and evaluated for chromium responsiveness by measuring cell proliferation after 5 days of culture. Data are presented as median values with 1st and 3rd quartiles, along with minimum and maximum values (Cr: n = 6; HC: n = 6). Statistical comparisons between groups (Cr vs. HC) for each parameter were conducted using Mann–Whitney U test, *** *p* < 0.001; **** *p* < 0.0001. Abbreviations: PBMC = peripheral blood mononuclear cells; CD4^+^ = helper T cells; CD8^+^ = cytotoxic T cells; CD25^+^ = cells that express surface marker CD25; Tγδ = gamma delta T Cells.

PBMCs and T cell subsets isolated from healthy individuals and patients allergic to chromium were analyzed for their proliferative response to chromium exposure (Figure 1). In healthy donors, significant proliferation was observed in the CD4^+^CD25^−^, CD8^+^CD25^−^, and Tγδ cell fractions in response to chromium, indicating the presence of responsive T cell populations in the absence of allergic disease. In contrast, in chromium-allergic patients, not only did CD4^+^CD25^−^, CD8^+^CD25^−^, and Tγδ^+^ cells exhibit a proliferative response, but the entire PBMC, CD4^+^, and CD8^+^ fraction also showed statistically significant proliferation. The activation of PBMCs in allergic subjects suggests the presence of an immunological mechanism that fails to adequately suppress CD4 and CD8 T cell responses. In each experiment none of the cells proliferated when no Cr added (blue boxes).

**Figure 2:**
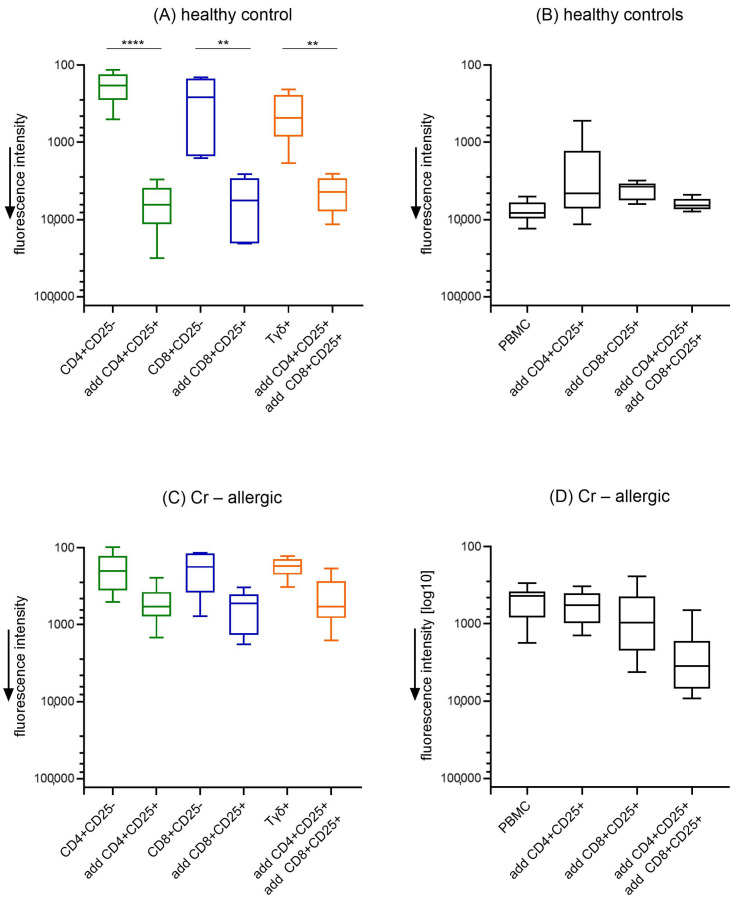
Modulatory effect of Treg cells on chromium-specific proliferation of PBMC subpopulations. CD4^+^, CD8^+^, Tγδ^+^, or whole PBMCs from (**A**,**B**) healthy controls and (**C**,**D**) chromium (Cr)-allergic individuals were cultured in vitro with chromium, with or without addition of Treg cells (50,000 of CD4^+^CD25^+^, CD8^+^CD25^+^, or both) to assess their potential inhibitory effect. Cr responsiveness was evaluated by measuring cell proliferation after 5 days of culture. Data are presented as median values with 1st and 3rd quartiles, along with minimum and maximum values (Cr: n = 6; HC: n = 6). Statistical comparisons between groups (Cr vs. HC) for each parameter were conducted using Mann–Whitney U test, ** *p* < 0.01; **** *p* < 0.0001. Abbreviations: PBMC = peripheral blood mononuclear cells; CD4^+^ = helper T cells; CD8^+^ = cytotoxic T cells; CD25^+^ = cells that express surface marker CD25; Tγδ = gamma delta T Cells.

To investigate whether regulatory cells (Treg) have the capacity to modulate Cr-specific T cell responses, we analyzed the proliferative response of Cr-specific T cell subsets in healthy controls and chromium-allergic individuals in the presence of CD4^+^CD25^+^ or CD8^+^CD25^+^ regulatory cells to assess the modulation of immune reactivity. In Figure 2, panel (A), healthy controls displayed significant proliferation within specific subsets (CD4^+^CD25^−^, CD8^+^CD25^−^, and Tγδ^+^ cells) in response to Cr exposure, while the addition of CD4^+^CD25^+^, CD8^+^CD25^+^, or both cells reduced proliferation, suggesting an inhibitory effect that may be caused by cytokine release or are cell contact-dependent. Panel (C) shows chromium-allergic individuals demonstrate sustained proliferation with no statistical decrease when Treg cells (CD4^+^CD25^+^, CD8^+^CD25^+^, or both) are added. This lack of suppression might suggest that, in Cr-allergic individuals, Treg cells fail to adequately modulate the proliferative response to Cr, indicating a potential impairment in regulatory function. This observation aligns with the findings in panel (D), where whole PBMCs from Cr-allergic individuals exhibit robust proliferation upon Cr exposure, and the addition of Treg cells (CD4^+^CD25^+^, CD8^+^CD25^+^, or both) fails to suppress this proliferative response. In contrast, panel (B) demonstrates that in healthy individuals, whole PBMCs do not show proliferation in response to chromium exposure, and the addition of Treg cells does not alter this non-proliferative state, underscoring the controlled immune tolerance to chromium in healthy subjects.

### 3.3. Cytokine Assessment

**Figure 3:**
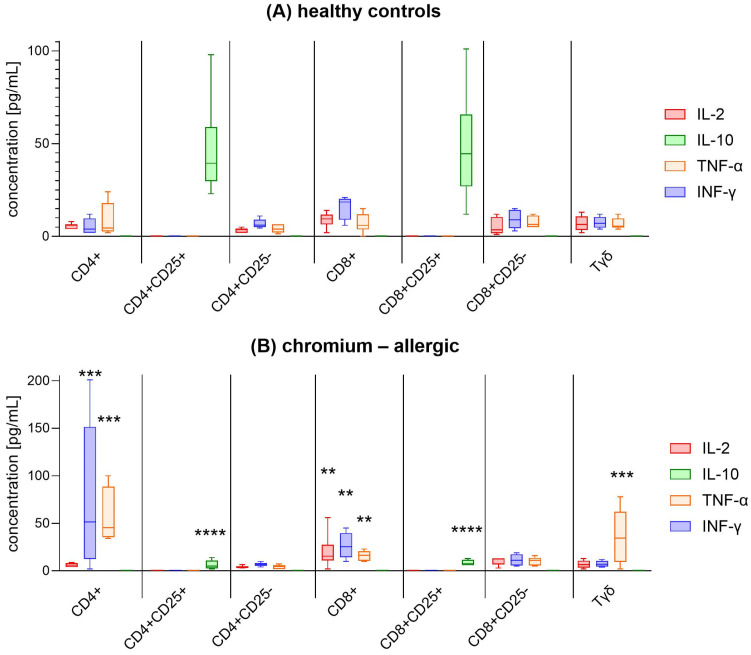
Cytokine release by chromium-specific proliferation of PBMC subpopulations. Different subpopulations isolated from PBMCs of healthy controls (HCs) and chromium-allergic individuals were assessed for cytokine release. Cytokine release was calculated after 5 days of culture with Cr. Data are presented as median values with 1st and 3rd quartiles, along with minimum and maximum values (Cr: n = 6; HC: n = 6). Statistical comparisons between groups (Cr vs. HC) for each parameter were conducted using Mann–Whitney U test, ** *p* < 0.01; *** *p* <0.001; **** *p* < 0.0001. Abbreviations: PBMC = peripheral blood mononuclear cells; CD4^+^ = helper T cells; CD8^+^ = cytotoxic T cells; CD25^+^ = cells that express surface marker CD25; Tγδ = gamma delta T Cells.

The cytokines assessed included IL-2, IL-4, IL-6, IL-10, TNF, IFN-γ, and IL-17A. However, detectable levels were observed only for IL-2, IFN-γ, TNF-α, and IL-10 (Figure 3). In Figure 3A, healthy controls show moderate pro-inflammatory cytokine release, with significant high production of IL-10 observed particularly in the CD4^+^CD25^+^ and CD8^+^CD25^+^ cells. IFN-γ release is observed at lower levels across several subsets; TNF-α and IL-2 are also produced but remain relatively low in most cell types. The presence of IL-10 in healthy individuals suggests an effective regulatory mechanism that may contribute to immune tolerance to chromium.

In contrast, Figure 3B reveals a distinct cytokine profile in chromium-allergic individuals. These allergic individuals show elevated levels of IL-2 in CD8^+^ cells, with significant statistical differences compared to healthy controls (** *p* < 0.01). IFN-γ production is highly elevated in the CD4^+^ cells (*** *p* < 0.001), and also increased in CD8^+^ cells, though to a lesser extent (** *p* < 0.01). TNF-α is elevated in CD4^+^ and Tγδ (*** *p* < 0.001). Importantly, IL-10 production is absent across all cell types in allergic individuals, which may indicate a failure of anti-inflammatory control in chromium allergy, possibly contributing to their hypersensitivity. In Cr-ACD patients, CD4^+^ T cells exhibited significantly elevated production of TNF-α and IFN-γ compared to the healthy controls. However, this increase was not observed in the CD4^+^CD25^−^ subset. A similar pattern was noted in the CD8^+^ and CD8^+^CD25^−^ subsets, where CD8^+^ T cells demonstrated increased pro-inflammatory cytokine production, but CD8^+^CD25^−^ cells did not exhibit a comparable rise. These findings suggest that regulatory dysfunction in ACD extends beyond IL-10 deficiency and involves additional immunomodulatory mechanisms, including direct cell-to-cell contact, which warrant further investigation.

### 3.4. Mitochondrial Activity

**Figure 4:**
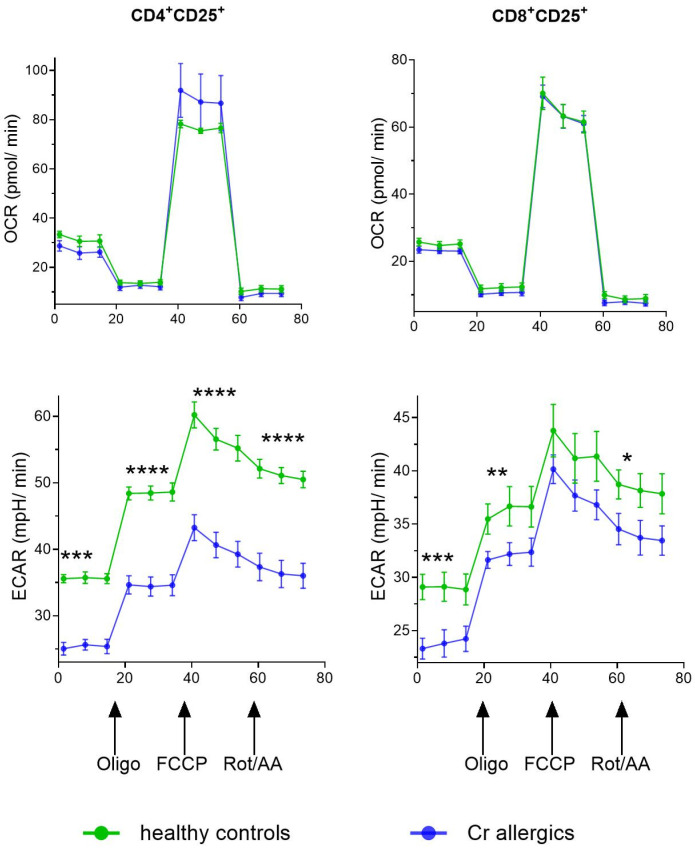
Mitochondrial activity. Oxygen consumption rate (OCR) and extracellular acidification rate (ECAR) were measured in 5-day in vitro cultures of Treg cell subpopulations (CD4^+^CD25^+^ and CD8^+^CD25^+^) from healthy controls and chromium-allergic individuals exposed to chromium (Cr). Assessments were conducted under basal conditions and following addition of mitochondrial inhibitors: oligomycin (Oligo), cyanide-4-(trifluoromethoxy)phenylhydrazone (FCCP), and combination of rotenone and antimycin A (Rot/AA). Results are presented as mean values with standard deviations (SD), with significance levels indicated as * *p* < 0.05; ** *p* < 0.01; *** *p* < 0.001; **** *p* < 0.0001.

Figure 4 illustrates mitochondrial activity in Treg subpopulations (CD4^+^CD25^+^ and CD8^+^CD25^+^) from healthy controls and chromium-allergic individuals after 5 days of in vitro culture with chromium. The oxygen consumption rate (OCR) remained relatively stable across both groups, indicating no significant differences in mitochondrial respiration between healthy and chromium-allergic Treg cells. In contrast, the extracellular acidification rate (ECAR) was consistently higher in healthy controls compared to chromium-allergic individuals, particularly following treatment with mitochondrial inhibitors. This increased ECAR suggests higher glycolytic activity in the Treg cells of the healthy controls, which may contribute to the regulatory function observed in these cells.

## 4. Discussion

Chromium is a potent skin sensitizer that persistently engages the immune system due to its widespread presence and ability to penetrate the skin barrier [29]. Consequently, many individuals, regardless of their allergic status, harbor circulating chromium-specific memory T cells. However, only certain individuals develop an allergic response to chromium, while others do not, suggesting the presence of regulatory mechanisms that determine susceptibility to chromium-induced hypersensitivity.

The data in Figure 1 highlights a distinct difference in chromium responsiveness between healthy controls and chromium-allergic participants. In healthy individuals, proliferation in response to chromium was restricted to specific T cell subpopulations, namely CD4^+^CD25^−^, CD8^+^CD25^−^, and Tγδ^+^ cells, suggesting a controlled immune response without broader activation. Conversely, in chromium-allergic individuals, a significantly higher proliferation was observed not only in the CD4^+^CD25^−^, CD8^+^CD25^−^, and Tγδ subsets but also across the entire PBMC population, including CD4^+^ and CD8^+^ cells. This broader activation in allergic individuals indicates a dysregulated immune response, likely due to an inability to suppress CD4^+^ and CD8^+^ T cell activation effectively. The lack of proliferation in cells without chromium exposure (blue boxes) in both groups confirms that the observed responses are chromium-specific.

In the setting of chromium allergy, our findings suggest a dysregulation of Tγδ cell function, where these cells may shift towards a predominantly inflammatory phenotype, contributing to heightened immune activation rather than immune tolerance. Previous studies have suggested that Tγδ cells can either exacerbate or dampen allergic responses depending on the presence of co-stimulatory signals and cytokine milieu [30,31]. Our data align with these findings by showing an increased activation of Tγδ cells in chromium-allergic individuals, further supporting their involvement in shaping immune responses in metal hypersensitivity. Given their plasticity, future research should focus on dissecting the specific Tγδ subsets involved in chromium allergy and their potential as therapeutic targets for modulating immune reactivity.

The observed data indicate a significant disparity in the regulatory capacity of T cells between healthy individuals and those with chromium (Cr) allergy. In healthy controls, Tregs cells effectively suppress the proliferation of Cr-reactive T cell subsets, maintaining immune tolerance. Conversely, in Cr-allergic patients, Tregs fail to inhibit this proliferation, suggesting a dysfunction in regulatory mechanisms. This impaired Treg function may contribute to the heightened hypersensitivity observed in these individuals. Similar findings were reported in other metal allergies, where defective Treg activity leads to increased effector T cell responses and allergic manifestations [32,33]. For example, a study on nickel allergy showed that regulatory T cells (particularly CD4^+^CD25^+^ cells) can effectively suppress nickel-specific T cell responses in healthy individuals, while this suppression fails in individuals with nickel allergy [34]. This pattern suggests that a comparable dysfunction in Treg-mediated suppression could underlie the heightened immune response in chromium-allergic patients, highlighting a broader role of regulatory T cell deficiencies across different metal allergies.

Moreover, recent evidence suggests that Treg dysfunction in metal allergies may be both cytokine-independent and cell contact-dependent, pointing to complex mechanisms by which Tregs interact with effector T cells to maintain tolerance [26,35,36,37,38,39]. This observation underscores the potential for targeted therapeutic approaches that could restore effective Treg function in chromium-allergic patients, thereby reducing hypersensitivity reactions and improving clinical outcomes. Our findings reveal distinct cytokine profiles in chromium-allergic individuals compared to healthy controls, with elevated IL-2 and IFN-γ levels and a lack of IL-10 in allergic patients [40]. This pattern suggests a heightened pro-inflammatory response and impaired regulatory mechanisms in chromium allergy. These results align with previous studies showing that IL-2 and IL-13 can effectively differentiate allergic patients from controls, supporting the potential of cytokine profiling as a diagnostic marker for chromium allergy. The absence of IL-10 further suggests a deficiency in anti-inflammatory regulation, contributing to hypersensitivity [41]. The absence of IL-10 in chromium-allergic individuals suggests a failure in key regulatory mechanisms essential for immune tolerance. IL-10 deficiency may contribute to heightened immune reactivity, reinforcing the pro-inflammatory cytokine milieu observed in allergic patients. This finding highlights the potential for IL-10 as a biomarker for chromium allergy and underscores the need for future therapeutic strategies aimed at restoring immune regulation, such as cytokine-based immunomodulation or enhancing regulatory T cell function.

Cellular processes such as activation, proliferation, and memory formation are heavily influenced by metabolic reprogramming. Mitochondrial respiration and glycolysis play crucial roles in sustaining these cellular functions [42]. The majority of intracellular adenosine triphosphate (ATP) is produced through glycolysis and mitochondrial oxidative phosphorylation. Real-time monitoring of these metabolic pathways in live cells provides dynamic, functional insights into cellular bioenergetics [43]. In our study, the stable OCR and elevated ECAR in the healthy controls suggest that enhanced glycolytic activity in Treg cells may contribute to their effective regulatory function, a balance disrupted in chromium-allergic individuals. These findings are supported by previous studies, demonstrating that mitochondrial dysfunction and oxidative stress impair Treg function, leading to immune dysregulation [44]. Furthermore, glycolysis-driven metabolic flexibility is necessary for optimal Treg activity [45]. Given that mitochondrial alterations were implicated in autoimmune diseases and chronic inflammatory conditions, our results suggest that metabolic dysfunction may be a key factor in Cr-ACD pathogenesis.

Our findings suggest that immune and metabolic profiling could improve the accuracy of chromium hypersensitivity diagnostics. The standard chromium patch test has limitations, including variability and false results. Our data indicate that immune markers such as chromium-specific T cell proliferation, IL-10 deficiency, and Treg metabolic dysfunction (reduced ECAR) could serve as complementary diagnostic indicators to better classify hypersensitivity phenotypes. This stratification could enhance diagnostic precision. Additionally, targeting Treg metabolism may offer a therapeutic strategy to restore immune tolerance, highlighting the need for more precise diagnostics and personalized treatment approaches.

This study has several limitations that should be addressed in future research. One significant limitation is the lack of direct analysis of whole blood. The study exclusively used isolated PBMCs, which do not fully replicate the complex physiological environment of whole blood, where factors such as plasma proteins and additional immune components could influence the immune response to chromium. While PBMC-based assays provide a controlled system for studying chromium-specific immune reactions, they do not fully capture the intricate cell–cell interactions, cytokine networks, and systemic regulatory mechanisms present in vivo. The next limitation of this study is that the proportion of CD25^+^ cells within the total CD4^+^ and CD8^+^ T cell populations was not determined. Future studies should address this limitation to clarify whether the proportion of CD25^+^ cells is altered in chromium-induced hypersensitivity. Another limitation is the absence of data from skin biopsies or in vivo analysis. Since chromium hypersensitivity primarily manifests in the skin, the lack of skin-specific data limits the study’s ability to correlate systemic immune responses with localized tissue pathology. Additionally, variability among human samples introduces another layer of complexity, as individual immune responses to chromium may differ based on genetic, environmental, and occupational factors. Addressing these gaps could provide a more comprehensive understanding of chromium-induced hypersensitivity.

A key limitation of this study is the small sample size, which may affect the statistical power and generalizability of the findings. To address this, we applied the non-parametric Mann–Whitney U test, which is suitable for small sample sizes and does not assume normal data distribution. While the statistical significance observed supports the identified trends, larger cohort studies are needed to validate these results and strengthen the conclusions.

## 5. Conclusions

The findings emphasize the importance of balanced interactions between effector, regulatory, and Tγδ cells in determining chromium tolerance or hypersensitivity. The selective proliferation of CD4^+^CD25^−^, CD8^+^CD25^−^, and Tγδ^+^ cells in healthy individuals suggests an immune response that is tightly regulated and confined to specific T cell subsets. In healthy controls, Tγδ cells may play a unique role in maintaining immune balance, possibly by responding to stress signals without triggering extensive inflammation. In contrast, chromium-allergic individuals exhibit broader activation, including heightened responses in both CD4^+^ and CD8^+^ cells, which points to the inability of regulatory cells to adequately control effector cell activation. The observed activation of Tγδ cells in allergic individuals further suggests that these cells, normally involved in early immune responses, may contribute to the dysregulated immune environment characteristic of chromium hypersensitivity. This imbalance highlights the role of Tregs in suppressing chromium-specific T cell responses and maintaining immune tolerance, while their dysfunction may contribute to the hypersensitivity observed in allergic patients.

Clinically, these insights underscore the potential for therapeutic interventions that target Treg and Tγδ cell function, offering a pathway to restore immune balance and reduce chromium hypersensitivity, thus improving outcomes for affected patients.

## Data Availability

All data are archived at the Department of Clinical Immunology (Medical University of Wroclaw, Poland) and can be provided by the author.

## Supplementary Materials

The following supporting information can be downloaded at: https://www.mdpi.com/article/10.3390/jcm14041370/s1, Figure S1: Representative example of flow cytometric gating strategy for PBMCs; Figure S2: Representative example of flow cytometric analysis of cytokine secretion; Figure S3: Verification of cell purification using MACS MicroBeads; Figure S4: Cytokine release by chromium-specific proliferation of PBMC subpopulations.
