# Supplementary material for: The Interaction Among Effector, Regulatory, and Tγδ Cells Determines the Development of Allergy or Tolerance to Chromium
Source: J Clin Med. 2025 Feb 19;14(4):1370. doi: 10.3390/jcm14041370 (PMC11856200; doi:10.3390/jcm14041370)
Supplement: Supplementary file 1 [file jcm-14-01370-s001.zip › jcm-3323130-supplementary.pdf]

SUPPLEMENTARY

FACSDiva Version 6.1.3

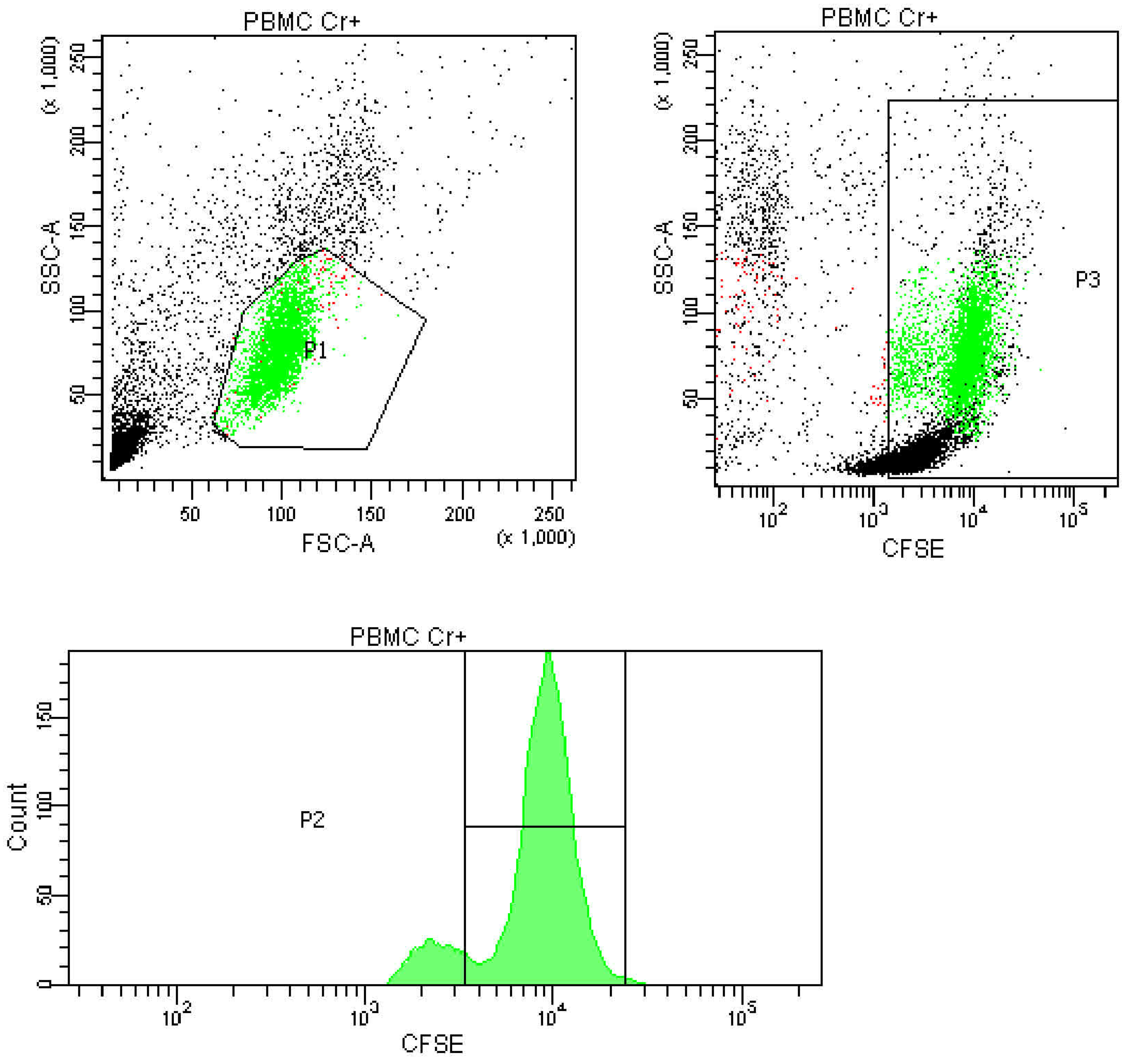

Experiment Name: chromium allergic - patient 1

Population	#Events	%Parent	Geo Mean	Mean	Median
P3	4,114	96.6	7,921	8,960	8,962

Tube: PBMC Cr+

Population	#Events	%Parent	%Total
All Events	10,000	####	100.0
P1	4,257	42.6	42.6
P3	4,114	96.6	41.1
P2	3,637	88.4	36.4

Figure S1. Representative example of flow cytometric gating strategy applied to peripheral blood mononuclear cells (PBMCs) derived from a patient with chromium allergy (stimulated with Cr). This figure illustrates the step-by-step gating process used to identify and analyze specific cell populations of interest. The gating is based on fluorescence intensity.

FACSDiva Version 6.1.3

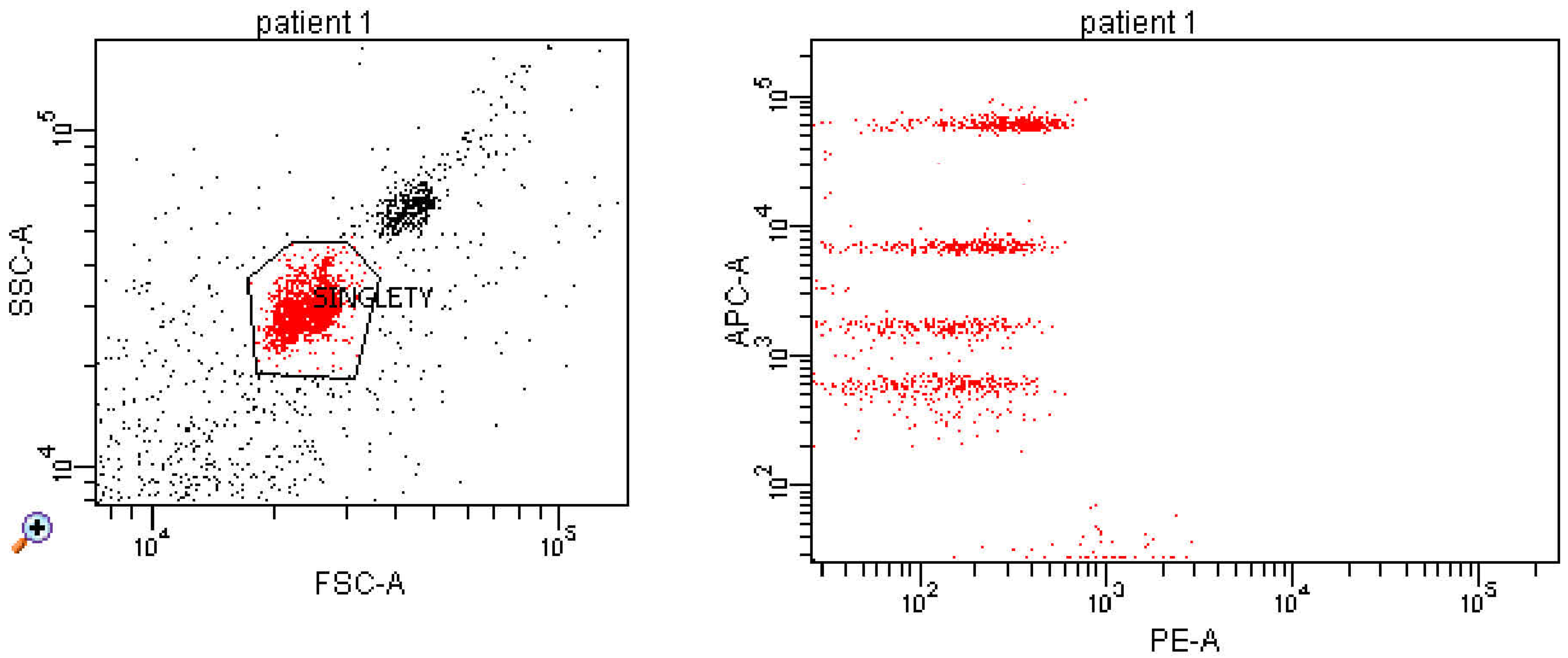

Tube: patient 1

Population	#Events	%Parent	%Total
All Events	3,649	####	100.0
SINGLETY	2,065	56.6	56.6

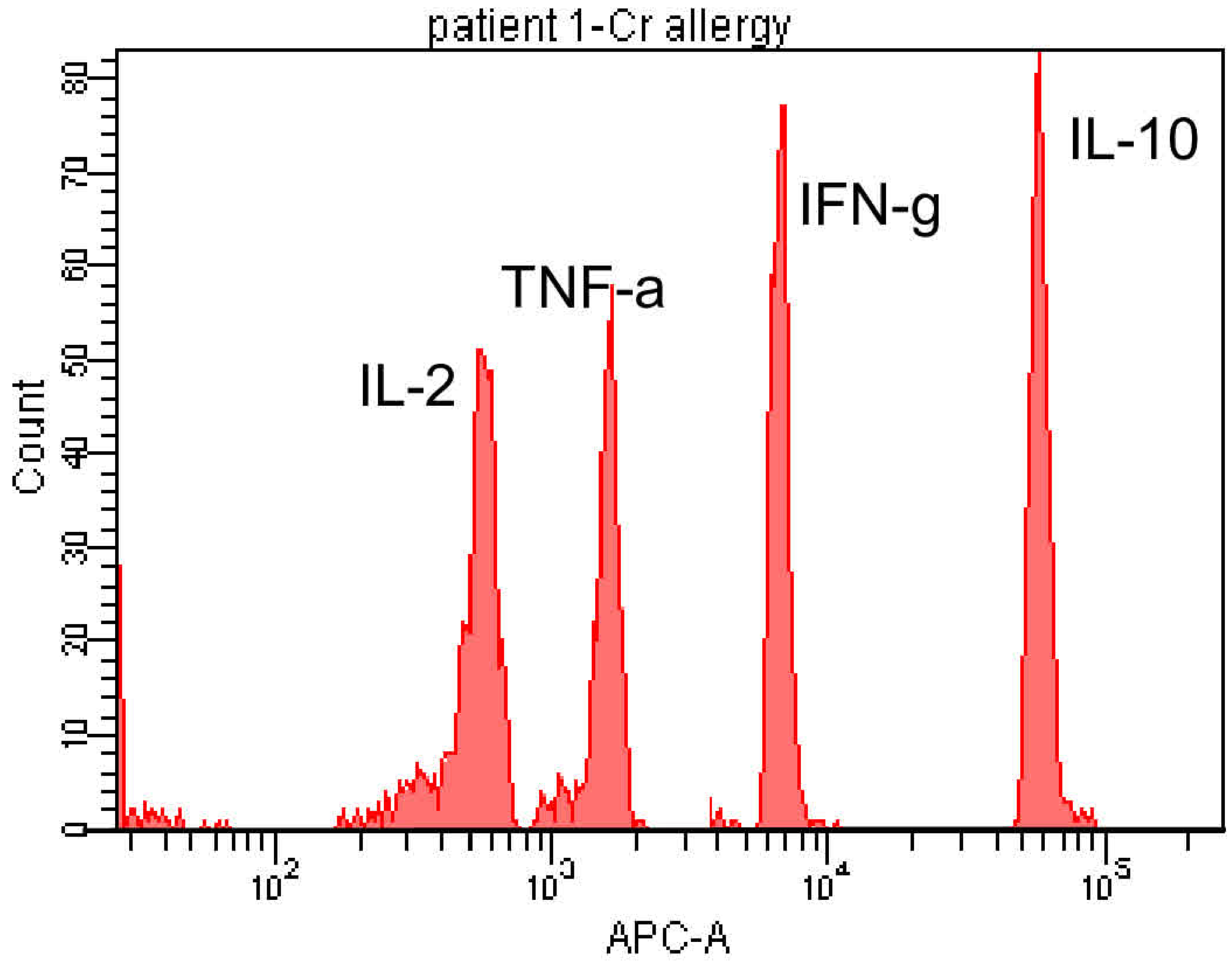

Figure S2. Representative example of flow cytometric analysis of cytokine secretion from a patient with chromium allergy (PBMC). Cytokine levels were quantified by comparing sample fluorescence intensities to standard curves generated using known cytokine concentrations (supplied by the manufacturer as part of the kit). The analysis was conducted with FCAP Array Software, specifically optimized for data obtained from BD Cytometric Bead Array (CBA) assays.

Figure S3. Verification of cell purification using Miltenyi Biotec MACS MicroBeads.

Flow cytometry analysis of cells after CD4+ separation from PBMCs and subsequent second CD25+ separation. (a) $CD4^{+}CD25^{+}$ cells obtained from positive selection using CD25 MACS MicroBeads. (b) $CD4^{+}CD25^{-}$ cells representing the negative fraction, left after positive selection. Purity assessment was performed to confirm the efficiency of magnetic separation.

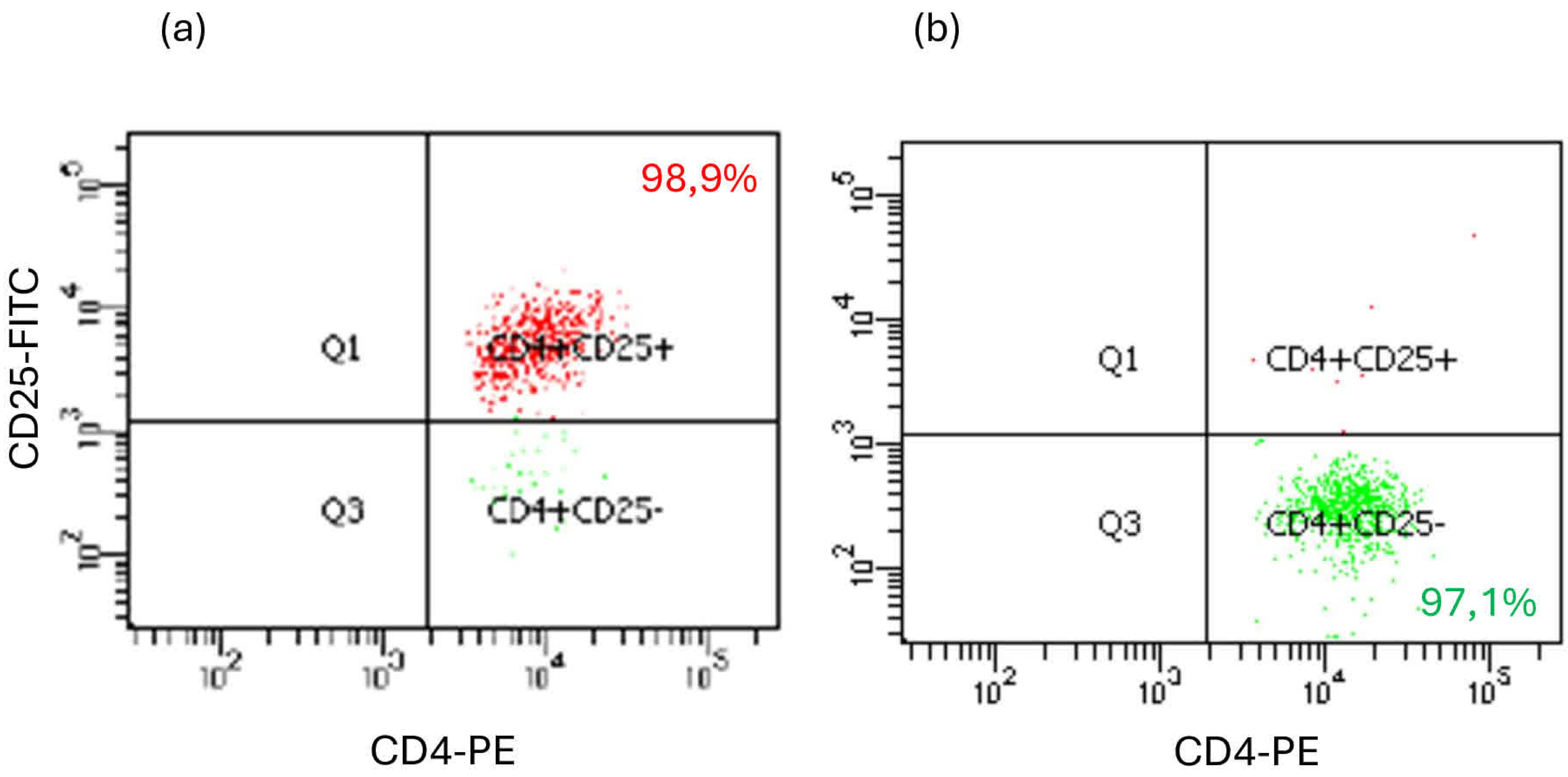

Figure S4. Cytokine release by chromium-specific proliferation of PBMC subpopulations.

A) Healthy controls

Row statistics	A			B			C			D		
	IL-2			INF-γ			TNF-α			IL-10		
	Median	Upper Limit	Lower Limit	Median	Upper Limit	Lower Limit	Median	Upper Limit	Lower Limit	Median	Upper Limit	Lower Limit
CD4+	6.00	8.00	4.00	4.00	12.00	2.00	4.50	24.00	2.00	0.00	0.00	0.00
CD4+CD25+	0.00	0.00	0.00	0.00	0.00	0.00	0.00	0.00	0.00	39.50	98.00	23.00
CD4+CD25-	3.75	5.00	2.00	6.15	11.00	4.60	3.95	6.50	1.40	0.00	0.00	0.00
CD8+	9.50	14.00	2.00	18.50	21.00	6.00	6.00	15.00	0.00	0.00	0.00	0.00
CD8+CD25+	0.00	0.00	0.00	0.00	0.00	0.00	0.00	0.00	0.00	44.50	101.00	12.00
CD8+CD25-	3.50	12.00	1.00	9.00	15.00	3.00	6.50	12.00	5.00	0.00	0.00	0.00
Tγδ	6.50	13.00	2.00	7.00	12.00	4.00	5.50	12.00	4.00	0.00	0.00	0.00

B) Chromium allergic

Row statistics	A			B			C			D		
	IL-2			INF-γ			TNF-α			IL-10		
	Median	Upper Limit	Lower Limit	Median	Upper Limit	Lower Limit	Median	Upper Limit	Lower Limit	Median	Upper Limit	Lower Limit
CD4+	6.00	9.00	4.00	51.50	201.00	2.00	45.50	100.00	34.00	0.00	0.00	0.00
CD4+CD25+	0.00	0.00	0.00	0.00	0.00	0.00	0.00	0.00	0.00	5.00	14.00	2.00
CD4+CD25-	3.85	6.60	3.00	6.95	9.80	4.20	4.45	7.50	2.00	0.00	0.00	0.00
CD8+	15.50	56.00	2.00	25.50	45.00	10.00	16.50	23.00	10.00	0.00	0.00	0.00
CD8+CD25+	0.00	0.00	0.00	0.00	0.00	0.00	0.00	0.00	0.00	8.00	13.00	6.00
CD8+CD25-	12.50	13.00	3.00	11.00	19.00	5.00	11.00	16.00	5.00	0.00	0.00	0.00
Tγδ	6.50	13.00	2.00	7.00	12.00	4.00	34.50	78.00	2.00	0.00	0.00	0.00
